# Long-Term Outcomes of Targeted Volume Overload Management in Patients With Severe Aortic Stenosis

**DOI:** 10.1016/j.jacadv.2026.102705

**Published:** 2026-04-01

**Authors:** Maximilian Autherith, Kseniya Halavina, Laurenz Hauptmann, Sophia Koschatko, Charlotte Jantsch, Gregor Heitzinger, Varius Dannenberg, Daryush Danesh, Caglayan Demirel, Christian Hengstenberg, Julia Mascherbauer, Philipp E. Bartko, Christian Nitsche

**Affiliations:** a Division of Cardiology, Department of Internal Medicine II, Medical University of Vienna, Vienna, Austria; b Department of Internal Medicine 3, University Hospital St. Poelten, Karl Landsteiner University of Health Sciences, Krems, Austria

**Keywords:** bioimpedance spectroscopy, decongestion, heart failure, TAVI

## Abstract

**Background:**

Patients with severe aortic stenosis (AS) and volume overload (VO) remain at an increased risk for heart failure and death after transcatheter aortic valve replacement (TAVR). The EASE-TAVR (Bioimpedance guided management of patients scheduled for transcatheter aortic valve replacement) trial demonstrated that decongestive treatment by bioimpedance spectroscopy (BIS) improves 1-year outcomes post-TAVR, whereas long-term effects are unknown.

**Objectives:**

This study aimed to investigate outcomes of BIS-guided decongestion in AS patients at 36 months after TAVR.

**Methods:**

EASE-TAVR randomized patients with severe AS and VO defined by BIS (≥1.0 L and/or ≥7%) 1:1 into: 1) BIS-guided decongestion (n = 55); or 2) decongestion by clinical judgment alone (n = 56) post-TAVR. Patients without VO (n = 121) served as the euvolemic control group. BIS-guided decongestion was performed for 12 months. The primary endpoint was a composite of heart failure hospitalization (HFH) and all-cause death at 36 months. The secondary endpoints included separate analyses of primary endpoint components and frequency of HFH.

**Results:**

Patients in the BIS-guided group had higher diuretic doses compared to the non–BIS-guided group throughout the entire study. At 36 months, the primary endpoint occurred in 21.8% in the BIS-guided and 46.4% in the non–BIS-guided group yielding a hazard reduction of 60% (HR: 0.40; 95% CI: 0.20-0.79). BIS-guided decongestion led to a reduced cumulative incidence of first HFH (3.6%; 95% CI: 0.7-11.2 vs 25.0%; 95% CI: 14.5-37.0), a reduced HFH frequency (28 vs 173 per 1,000 patient years; *P* < 0.001), and showed a nonsignificant reduced mortality hazard (HR: 0.54; 95% CI: 0.26-1.14) compared to decongestion as per clinical judgment alone.

**Conclusions:**

In patients with severe AS and VO, outcome benefits of BIS-targeted decongestive treatment persist up to 3 years after TAVR.

Aortic valve replacement (AVR) via surgical (SAVR) or transcatheter (TAVR) approach currently represents the only effective treatment for patients with severe aortic stenosis (AS).^1^ However, the sequelae of long-standing pressure overload and subsequent myocardial damage induced by AS subject patients to an increased hazard for heart failure and death despite successful AVR.^2^, ^3^, ^4^, ^5^ Volume overload (VO) represents an indicator for heart failure and can be objectively quantified using bioimpedance spectroscopy (BIS), overcoming the limitation of subjectivity and inaccuracy of clinical VO assessment. VO by BIS has been demonstrated to be associated with the extent of myocardial damage, multiorgan dysfunction, and with worse post-TAVR outcomes in AS.^5^, ^6^, ^7^, ^8^

Results from these observational studies nurtured the hypothesis that targeted decongestive treatment guided by BIS could have the potential to improve post-TAVR outcomes. Therefore, the randomized controlled EASE-TAVR trial (Bioimpedance guided management of patients scheduled for transcatheter aortic valve replacement) was designed to investigate the effectiveness of targeted decongestion compared to decongestion by clinical judgment alone in post-TAVR care (NCT04556123). BIS-guided decongestion led to a significant improvement in quality of life and reduction in heart failure hospitalization (HFH) and/or death at 1-year post-TAVR.^9^ Even though these results are promising and leverage rigorous fluid removal as a treatment pillar in post-TAVR management, the long-term outcomes of targeted decongestion in AS are unknown.

The aim of this analysis was to investigate clinical outcomes of patients enrolled in the EASE-TAVR trial through 36 months. We hypothesized that benefits achieved through intensified and targeted decongestive treatment would persist up to 36 months.

## Methods

### The EASE-TAVR trial

EASE-TAVR was a dual-center, prospective, randomized, controlled clinical trial investigating the effects of BIS-guided decongestive treatment in patients with AS. The trial design was prespecified (ClinicalTrails.gov Identifier: NCT04556123) and approved by the ethics committees of all participating sites (Medical University of Vienna: EK no. 2074/2019; University Hospital St. Poelten: EK no. 1050/2021). All patients provided written informed consent, and investigations were conducted according to the Declaration of Helsinki.^9^

A detailed methodological description of the EASE-TAVR trial has previously been reported.^9^ In brief, consecutive patients with severe AS scheduled for TAVR were eligible for enrollment and received VO assessment by BIS. Patients with VO according to BIS measurement on pre-TAVR assessment^5^^,^^7^^,^^10^ were randomly assigned to receive either a BIS-guided decongestion treatment or decongestion treatment as per clinical judgment alone. Subsequently, treating physicians were: 1) informed about BIS-measurement results and encouraged to adjust the diuretic management according to individualized measurement results (BIS-guided group); or 2) kept blinded to BIS measurements (non–BIS-guided group). Patients without VO on pre-TAVR assessment were included in the study and served as a euvolemic control group. Medication was collected at the time of randomization and before discharge from the hospital (post-TAVR). Within the 12-month follow-up period after TAVR randomized patients attended 2 on-site visits (day 90 ± 7; day 360 ± 7). In addition, 1 telephone visit (day 180 ± 7) and phone follow-ups as required for safety purposes were performed. The primary endpoint was a composite of HFH and/or all-cause death at month 12.^9^ Patients returned for yearly on-site follow-up visits after the first year post-TAVR.

### Transcatheter aortic valve replacement

Transthoracic echocardiography and TAVR were performed by board-certified cardiologists in accordance with international guidelines and with validated commercially available devices. Further details have previously been described.^5^^,^^9^^,^^11^^,^^12^ The decision to perform TAVR for AS treatment was performed by a multidisciplinary heart team.^9^^,^^11^^,^^12^ As study enrollment was performed during the TAVR-preparation period, reconsideration of heart team decision or patient preference led to changes in AS management strategies (SAVR, conservative management) in 11 cases. All randomized patients were, nevertheless, included and received trial interventions and follow-up according to the study protocol.^9^

### Bioimpedance spectroscopy and decongestive treatment

Assessment of VO was conducted by using a portable BIS device (Body Composition Monitor; Fresenius Medical Care), which allows inexpensive and noninvasive measurement of absolute VO (in liters [l]) and relative VO (%). Further details on correct measurements and principles of BIS have previously been described.^5^^,^^7^^,^^9^^,^^13^ In accordance with previously published data, significant VO was defined as absolute VO ≥1.0 L and/or relative VO ≥7% in the pre-TAVR BIS assessment.^5^^,^^7^^,^^9^^,^^10^ The selection of diuretic agents for decongestive treatment remained at the discretion of the treating physician and was conducted in accordance with local standards. Further details on diuretic management have previously been described.^9^

### The long-term extension study

The present long-term extension study reports clinical outcomes at 36 months after TAVR. Following the final study visit at 12 months after TAVR, all patients were discharged to clinical routine follow-up for continued monitoring and treatment in accordance with local standards. Patients in the BIS-guided group were informed about their BIS-determined dry body weight and instructed to adapt diuretic treatment in consultation with their treating physician to maintain euvolemia. Patients in the non–BIS-guided group continued to be blinded with respect to BIS results. On-site follow-up visits, as part of clinical routine, were conducted at 24 and 36 months, with no further BIS-assessment or targeted decongestive treatment interventions performed by the study team (Supplemental Figure 1). Data on diuretic management and heart failure treatment were collected for all patients attending on-site visits. Mortality data were obtained via retrieval review from the Austrian Death Registry. Data on HFH were collected via systematic research of electronic hospital information systems of the Vienna General Hospital, the Vienna Health Care Association, and the national database for electronic health records, allowing assessment of all HFH in public hospitals in Austria. In addition, HFH abroad were recorded via phone follow-up. Endpoint adjudication regarding HFH was performed by an independent adjudication committee consisting of 2 assessors blinded with respect to group allocation. HFH was defined as a primary admission for heart failure and/or the necessity of intravenous diuretic treatment. Agreement between independent assessors was 100% concordant and outcome data at 36 months of follow-up was 100% complete.

### Clinical outcomes

The primary endpoint of this long-term extension study was defined as a composite of HFH and/or all-cause death over a period of 36 months. The secondary endpoints were: 1) time to all-cause death after 36 months; 2) time to HFH after 36 months; and 3) frequency of HFH after 36 months. To separate intervention and maintenance effects, landmark analyses at 12 months were conducted for the primary and secondary endpoints.

### Statistical methods

Continuous data are reported as mean ± SD or median with 25th–75th percentiles (Q1-Q3) based on distribution. Categorical variables are expressed as counts and percentages of the respective group. All differences between groups regarding continuous data are examined using the Wilcoxon rank-sum test, whereas differences among multiple groups used the Kruskal-Wallis test. For all group differences regarding categorical variables the chi-square or Fisher exact test was used. For the primary endpoint, all-cause death and/or HFH, and all-cause death alone, point estimates from Cox proportional hazards models are presented as HRs with respective 95% CIs. Group differences are analyzed using the log-rank test. To account for death as a competing risk, time to first HFH is assessed using cumulative incidence functions and is presented as percentage including respective 95% CIs. Group differences are analyzed using the Gray test. In addition, prespecified landmark analyses from month 12 to 36 were performed for patients still at risk at month 12 using similar models. The proportional hazards assumption was assessed using Schoenfeld residuals. Furthermore, absolute risk reduction (ARR), number needed to treat (NNT), and their respective 95% CIs are provided. If the 95% CI of the ARR included zero, the upper bound of the NNT was considered undefined. HFH frequency is expressed as events per 1,000 patient-years and analyzed both descriptively and, to account for differing follow-up times and recurrent events, with a negative binomial regression model including log-transformed follow-up time as an offset. Model-based HFH rates, incidence rate ratios (IRR) with 95% CIs and corresponding *P* values are reported. All analyses were primarily performed on the intention-to-treat principle, regardless of patients receiving TAVR, SAVR, or conservative management, as well as regardless of adherence to trial interventions and visits. Prespecified per-protocol analyses restricted to patients receiving TAVR are conducted as sensitivity analyses. Statistical analyses were performed using R (version 4.5.1, R Core Team) and IBM SPSS Statistics (version 29).

## Results

A total of 232 patients were enrolled in EASE-TAVR from September 2020 to January 2022. Patients with VO (n = 111) were randomized into the BIS-guided group (n = 55) and into the non–BIS-guided group (n = 56). Patients without VO (n = 121) served as the euvolemic control cohort (Central Illustration). All patients were scheduled for TAVR, but reconsideration of the heart team decision or patients’ preference eventually yielded conservative management in 10 patients (5 euvolemic group, 4 non–BIS-guided group, 1 BIS-guided group) and SAVR in 1 patient (BIS-guided group).

**Central Illustration:**
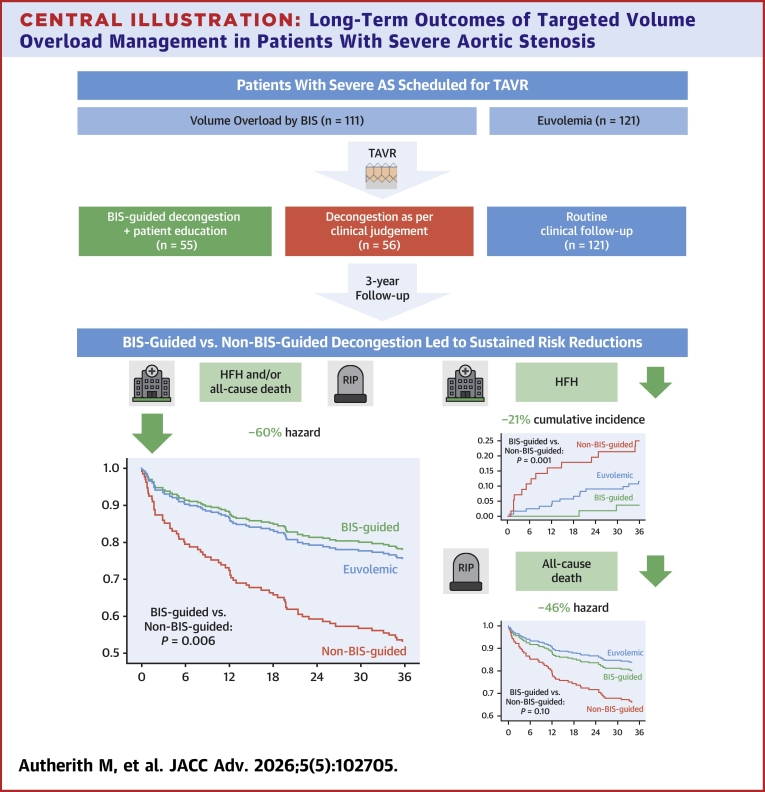
**Long-Term Outcomes of Targeted Volume Overload Management in Patients With Severe Aortic Stenosis** In patients with severe AS and volume overload, outcome benefits of BIS-targeted decongestive treatment on a composite of HFH and/or all-cause death persist up to 3-years after TAVR. In addition, a substantial cumulative incidence reduction in HFH, and a nonsignificant mortality reduction was observed at 3 years post-TAVR. AS = aortic stenosis; BIS = bioimpedance spectroscopy; HFH = heart failure hospitalization; TAVR = transcatheter aortic valve replacement.

### Patient characteristics

A summary of the baseline characteristics of patients is presented in Table 1. Comorbidities and other risk factors were well balanced between decongested patients in the BIS-guided vs non–BIS-guided group. Patients presented at a comparable disease stage as highlighted by comparable operative risk scores (EuroScore-II: 4.2% [Q1-Q3: 3.9-4.9] vs 4.3% [Q1-Q3: 3.9-4.8]), cardiac biomarkers (N-terminal pro-B-type natriuretic peptide: 2,033 pg/mL [Q1-Q3: 924-5,087] vs 2,058 pg/mL [Q1-Q3: 1,089-5,225]), clinical signs of congestion (any sign: 78.2% vs 66.7%), presence of atrioventricular valve incompetency on echocardiography (mitral regurgitation ≥ moderate: 28.6% vs 34.9%; tricuspid regurgitation ≥ moderate: 33.3% vs 31.8%), and quantitative VO measured by BIS (absolute VO: 1.8 L [Q1-Q3: 1.3-2.6] vs 2.3 L [Q1-Q3: 1.4-3.1]; Table 1). In addition, established heart failure medication was distributed equally between patients in the BIS-guided and non–BIS-guided group (renin-angiotensin system inhibitor: 60.0% vs 66.1%, mineralocorticoid receptor antagonist: 38.2% vs 33.9%, beta-blocker: 58.2% vs 60.7%, sodium-glucose co-transporter 2 inhibitor: 18.2% vs 8.9%) (Table 1), as was diuretic treatment at baseline (any diuretic drug: 66.0% vs 67.9%; loop diuretic: 45.5% vs 57.1%; thiazide: 20.5 vs 7.1) (Supplemental Table 1). Conversely, implemented diuretic medication after randomization (any diuretic drug: 100.0% vs 67.9%, *P* < 0.001; loop diuretic: 94.5% vs 57.1%, *P* < 0.001; thiazide: 12.7% vs 7.1%, *P* = 0.32) (Table 1) was more prevalent in the BIS-guided vs the non–BIS-guided group. Accordingly, in the BIS-guided group, furosemide-equivalent doses were higher compared to the non–BIS-guided group at all postrandomization study visits including the 12-month follow-up visit and the 24-month and 36-month visits during the long-term extension period (12 months: 33 ± 27 mg vs 21 ± 31 mg; 24 months: 30 ± 31 mg vs 19 ± 19 mg; 36 months: 32 ± 39 mg vs 12 ± 14 mg; all *P* < 0.05) (Table 2). Patients without VO presented at a less advanced stage of disease with lower operative risk scores, lower cardiac biomarkers, less pronounced clinical signs of congestion, and less pronounced cardiac damage markers on echocardiography compared to patients with VO (Table 1).

**Table 1:** Patient Characteristics Predischarge/Post-TAVR

Parameter	Missing Values	Volume Overload (N = 111)		No Volume Overload/Euvolemic Control Group (n = 121)
		BIS-Guided Group (n = 55)	Non–BIS-Guided Group (n = 56)	
Demographics				
Age, y	0/232	80.4 (78.5-83.5)	81.5 (78.9-85.3)	80.7 (75.4-83.4)
Female, n (%)	0/232	23 (41.8)	24 (42.9)	58 (47.9)
BMI, kg/m^2^	0/232	25.4 (22.4-28.0)^a^	25.8 (23.3-28.5)	27.7 (24.6-29.9)
Clinical parameters				
EuroSCORE-II, %	0/232	4.2 (3.9-4.9)^a^	4.3 (3.9-4.8)	4.1 (3.9-4.4)
KCCQ score, pts.	0/232	41 (31-52)	40 (31-52)	44 (35-54)
Hypertension, n (%)	0/232	45 (81.8)	53 (94.6)	111 (91.7)
Diabetes mellitus, n (%)	0/232	27 (49.1)	23 (41.1)	42 (34.7)
Atrial fibrillation, n (%)	0/232	21 (38.2)	28 (50.0)	39 (32.2)
CAD, n (%)	0/232	28 (50.9)	29 (51.8)	58 (47.9)
COPD, n (%)	0/232	7 (12.7)	6 (10.7)	11 (9.1)
Laboratory results				
eGFR, mL/min/1.73 m^2^	0/232	61 (47-83)	63 (42-74)	65 (49-79)
NT-proBNP, pg/mL	0/232	2,033 (924-5,087)^a^	2,058 (1,089-5,225)	990 (435-1861)
Symptoms				
Asymptomatic, n (%)	0/232	3 (5.5)	6 (10.9)	22 (18.2)
Dyspnea, n (%)	0/232	51 (92.7)	44 (78.6)	97 (80.2)
Chest pain, n (%)	0/232	13 (23.6)	18 (32.1)	31 (25.6)
Syncope, n (%)	0/232	10 (18.2)	15 (26.8)	27 (22.3)
Heart failure medication				
Any RAS inhibitor, n (%)	1/232	33 (60.0)	37 (66.1)	70 (58.3)
ACE inhibitor, n (%)	1/232	16 (29.1)	11 (19.6)	34 (28.3)
ARB, n (%)	1/232	14 (25.5)	22 (39.3)	34 (28.3)
ARNI, n (%)	1/232	3 (5.5)	4 (7.1)	2 (1.7)
MRA, n (%)	0/232	21 (38.2)	19 (33.9)	33 (27.3)
Beta-blocker, n (%)	1/232	32 (58.2)	34 (60.7)	69 (57.5)
SGLT2 inhibitor, n (%)	3/232	10 (18.2)	5 (8.9)	21 (17.8)
Diuretic medication				
Any diuretic drug, n (%)	0/232	55 (100.0)^a^	38 (67.9)	60 (49.6)
Loop diuretic, n (%)	0/232	52 (94.5)^a^	32 (57.1)	46 (38.0)
Thiazide, n (%)	0/232	7 (12.7)	4 (7.1)	4 (3.3)
Clinical signs of congestion				
Any sign, n (%)	0/232	43 (78.2)^a^	37 (66.1)	40 (33.1)
Chest x-ray, n (%)	52/232	8 (16.7)	2 (4.9)	5 (5.5)
Leg edema, n (%)	1/232	36 (65.5)^a^	33 (60.0)	37 (30.6)
Rales, n (%)	3/232	0 (0.0)^a^	4 (7.5)	0 (0.0)
Volume overload by BIS, L	0/232	1.8 (1.3-2.6)^a^	2.3 (1.4-3.1)	−0.1 (−0.9 to 0.4)
Aortic valve				
AV PPG, mm Hg	0/232	67 (56-84)	70 (54-81)	67 (57-81)
AV MPG, mm Hg	0/232	44 (35-53)	43 (34-51)	41 (36-50)
AV Vmax, m/s	0/232	4.1 (3.8-4.6)	4.2 (3.7-4.6)	4.1 (3.8-4.5)
Cardiac structure and function				
LVEF, %	0/232	57 (47-65)	59 (50-69)	62 (53-70)
LV mass index, g/m^2^	0/232	139 (113-168)^a^	143 (119-171)	126 (111-148)
LA volume index, mL/m^2^	0/232	43 (38-57)^a^	46 (41-54)	41 (35-48)
MR ≥ moderate, n (%)	48/232	14 (28.6)^a^	15 (34.9)	14 (15.2)
TR ≥ moderate, n (%)	46/232	16 (33.3)^a^	14 (31.8)	17 (18.1)
RV dysfunction ≥ moderate, n (%)	41/232	4 (8.0)	5 (11.4)	5 (5.2)

Categorical variables are presented as n (%) and continuous variables are reported as median (25–75th percentile).

ACE = angiotensin converting enzyme; ARB = angiotensin II receptor blocker; ARNI = angiotensin receptor neprilysin inhibitor; AV = aortic valve; BIS = bioimpedance spectroscopy; BMI = body mass index; CAD = coronary artery disease; COPD = chronic obstructive pulmonary disease; eGFR = estimated glomerular filtration rate; EuroSCORE = European System for Cardiac Operative Risk Evaluation; KCCQ = Kansas City Cardiomyopathy Questionnaire; LA = left atrium; LV = left ventricle; LVEF = left ventricular ejection fraction; MPG = mean pressure gradient; MR = mitral regurgitation; MRA = Mineralocorticoid receptor antagonist; NT-proBNP = N-terminal pro-B-type natriuretic peptide; PPG = peak pressure gradient; RAS = renin angiotensin system; RV = right ventricle; SGLT2 = sodium glucose co-transporter 2; TR = tricuspid regurgitation; Vmax = maximum velocity.

a Indicates *P* < 0.05 for comparison of no-FO-patients (euvolemic control group) vs FO-patients (combined BIS-guided and non–BIS-guided group).

**Table 2:** Heart Failure and Diuretic Medication During Follow-Up

Medical Treatment	BIS-Guided Group (n = 55)					No–BIS-Guided Group (n = 56)				
	Predischarge (n = 55, 100.0%)	3 Mo (n = 51, 100.0%)	12 Mo (n = 48, 100.0%)	24 Mo (n = 32, 69.6%)	36 Mo (n = 30, 68.1%)	Predischarge (n = 56, 100.0%)	3 Mo (n = 50, 100.0%)	12 Mo (n = 44, 100.0%)	24 Mo (n = 31, 79.5%)	36 Mo (n = 26, 70.3%)
HF-medication										
Any RAS inhibitor, %	60.0	84.1	75.8	85.5	72.7	37 (66.1)	77.8	75.0	82.9	80.0
ACE inhibitor, %	29.1	41.9	39.4	37.5	33.3	11 (19.6)	37.0	37.5	38.7	30.8
ARB, %	25.5	37.2	30.3	35.5	33.3	22 (39.3)	34.8	32.5	38.5	40.0
ARNI, %	5.5	4.7	6.1	12.5	13.3	4 (7.1)	6.5	5.0	15.4	15.0
MRA, %	38.2	51.1	40.0	46.9	56.7	19 (33.9)	48.8	25.7	45.2	53.8
Beta-blocker, %	58.2	58.1	66.7	56.3	53.3	34 (60.7)	76.1	76.9	74.2	69.2
SGLT2 inhibitor, %	18.2	18.6	24.2	28.1	33.3	5 (8.9)	15.2	20.0	29.0	38.5
Diuretic medication										
Loop diuretic, %	94.5^a^	73.3^a^	65.7^a^	62.5	63.3	32 (57.1)	48.8	45.7	48.3	50.0
Thiazide, %	12.7	11.1	15.6	19.2	16.7	4 (7.1)	14.0	17.1	11.5	15.0
Furosemide equivalent dose, mg	54 ± 34^a^	32 ± 32^a^	33 ± 27^a^	30 ± 31^a^	32 ± 39^a^	24 ± 29	22 ± 36	21 ± 31	19 ± 19	12 ± 14

HF = heart failure; other abbreviations as in Table 1.

Categorical variables are presented as % and continuous variables are reported as mean ± SD.

a Indicates *P* < 0.05 for comparison of BIS-guided vs non–BIS-guided group.

### Primary endpoint

**Figure 1:**
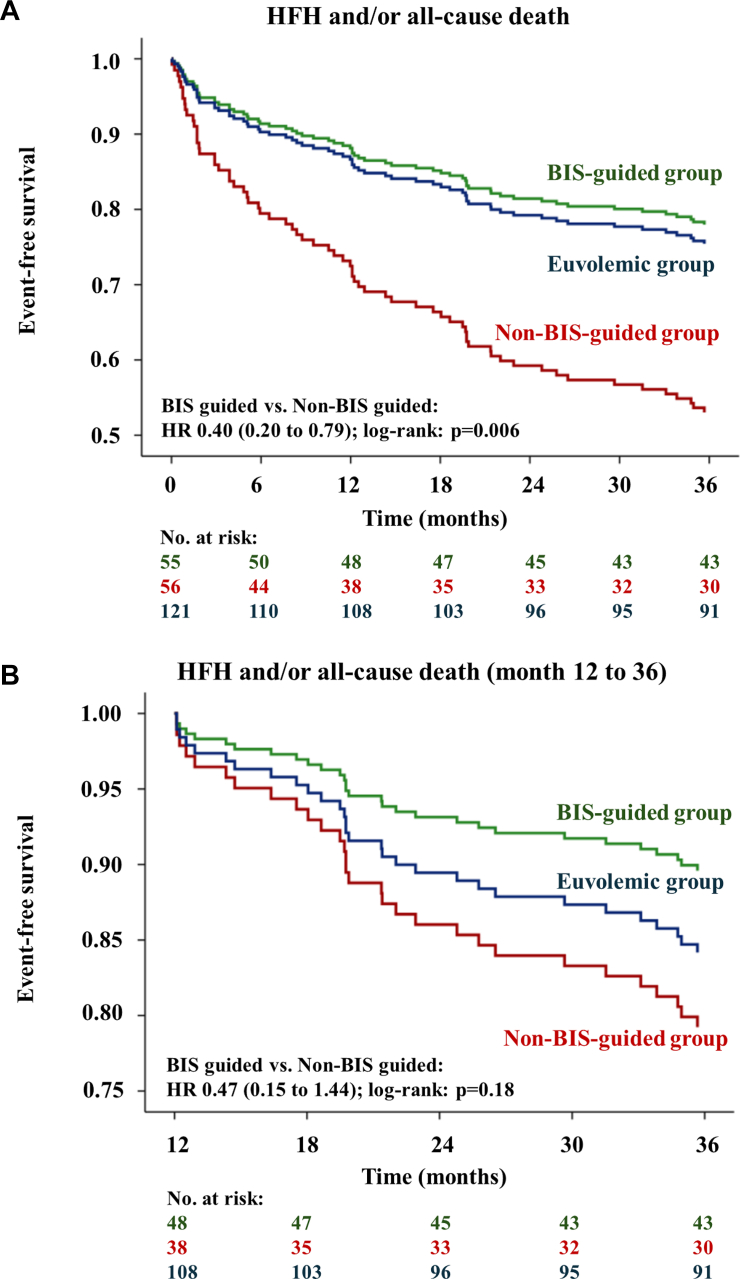
**Kaplan-Meier Estimates Illustrating Differences in the Primary Endpoint** (A) Kaplan-Meier estimates demonstrating a 60% reduced hazard of the primary endpoint, a composite of all-cause death and/or HFH, through month 36 and (B) a nonsignificant hazard reduction in the landmark analysis at month 12 for BIS-guided vs non-BIS-guided patients. BIS = bioimpedance spectroscopy; HFH = heart failure hospitalization.

At 36 months, 29.3% (68 of 232 patients) had experienced HFH or had died. Event rates according to study groups were 21.8% (12 of 55 patients) in the BIS-guided group, 46.4% (26 of 56 patients) in the non–BIS-guided group, and 24.8% (30 of 121 patients) in the euvolemic control group. BIS-guided decongestion was associated with a 60% reduced hazard for HFH and/or all-cause death compared to decongestion as per clinical judgment alone (HR: 0.40; 95% CI: 0.20-0.79; log-rank: *P* = 0.006) (Figure 1A) equaling an ARR of 24.6% (95% CI: 7.5-41.7) and a NNT of 4 (95% CI: 3-13). These results remained consistent after adjustment for the number of concomitant heart failure drug classes (adjusted HR: 0.41; 95% CI: 0.21-0.81) (Supplemental Table 2) and in the per-protocol population (HR: 0.42; 95% CI: 0.21-0.87) (Supplemental Table 3).

In the interventional period until 12-months, BIS-guided decongestion was associated with a 64% reduced hazard for HFH and/or all-cause death (HR: 0.36; 95% CI: 0.15-0.87). In a landmark analysis at 12-months, the mean event-free survival time was 22.6 months (95% CI: 21.2-23.9) in the BIS-guided group compared to 21.3 months (95% CI: 19.3-23.4) in the non–BIS-guided group. An ongoing nonsignificant 53% reduced hazard for HFH and/or all-cause death (HR: 0.47; 95% CI: 0.15-1.44; log-rank: *P* = 0.18) (Figure 1B) was observed, equaling an ARR of 10.7% (95% CI: -4.9% to 26.4%) and a NNT of 10 (95% CI: 4-undefined). These results remained consistent after adjustment for the number of concomitant heart failure drug classes (adjusted HR: 0.45; 95% CI: 0.14-1.39; log-rank: *P* = 0.16) and in the per-protocol population (HR: 0.55; 95% CI: 0.17-1.72; log-rank: *P* = 0.30).

Exploratory analysis demonstrated comparable outcomes of patients in the BIS-guided decongestion group and the euvolemic control group (HR: 0.94; 95% CI: 0.67-1.31; log-rank: *P* = 0.71). Conversely, patients in the non–BIS-guided group had worse outcomes than patients in the euvolemic control group (HR: 2.27; 95% CI: 1.33-3.85; log-rank: *P* = 0.002) (Figure 1A).

### Secondary endpoints

#### All-cause mortality

**Figure 2:**
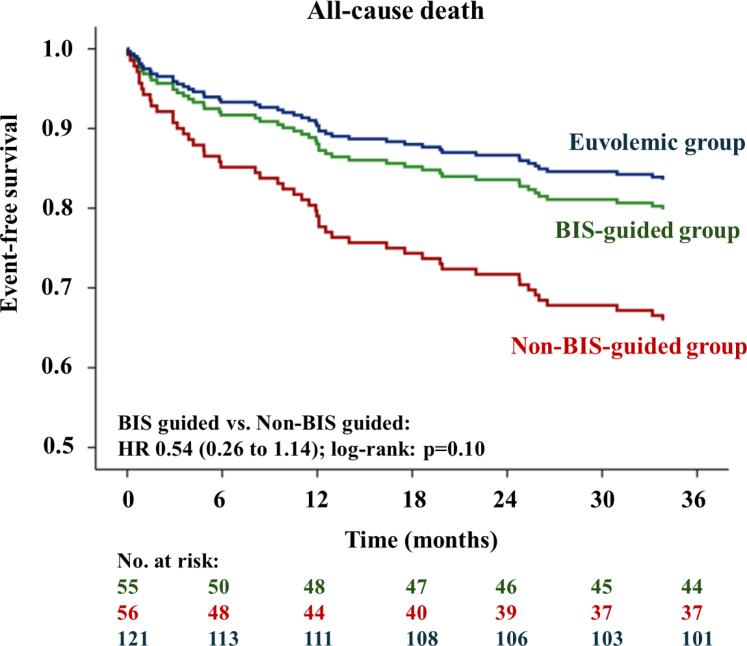
**Kaplan-Meier Estimates Illustrating Differences in All-Cause Mortality** Kaplan-Meier estimates demonstrating a nonsignificant 46% reduced hazard of all-cause death at month 36 for BIS-guided vs non–BIS-guided patients. Abbreviation as in Figure 1.

At month 36, across all 3 groups 21.6% (50 of 232 patients) had died; 20.0% (11 of 55 patients) in the BIS-guided group, 33.9% (19 of 56 patients) in the non–BIS-guided group and 16.5% (20 of 121 patients) in the euvolemic group. BIS-guided decongestive treatment showed a nonsignificant 46% reduced hazard of all-cause death compared to decongestion as per clinical judgment alone (HR: 0.54; 95% CI: 0.26-1.14; log rank: *P* = 0.10) (Figure 2), equaling an ARR of 13.9% (95% CI: −2.4% to 30.2%) and a NNT of 8 (95% CI: 4-undefined). These results remained consistent in the per-protocol population (HR:: 0.26-1.25; log-rank: *P* = 0.16) (Supplemental Table 3). In the interventional period until 12-months, BIS-guided decongestion did not provide convincing evidence for a reduced mortality risk (HR: 0.57; 95% CI: 0.22-1.45). In a landmark analysis at 12 months, the mean event-free survival time was 23.0 months (95% CI: 21.7-24.0) in the BIS-guided group compared to 21.7 months (95% CI: 19.9-23.6) in the non–BIS-guided group. The mortality risk was numerically lower in the BIS-guided group without statistical significance (HR: 0.57; 95% CI: 0.16-2.03; log rank: *P* = 0.39) equaling an ARR of 7.6% (95% CI: −5.7% to 20.9%) and a NNT of 14 (95% CI: 5-undefined).

Exploratory analysis revealed comparable risk for patients in the BIS-guided decongestion group and patients in the euvolemic control group (HR: 1.25; 95% CI: 0.60-2.61; log rank: *P* = 0.55). Conversely, patients in the non–BIS-guided group had higher mortality than patients in the euvolemic control group (HR: 2.33; 95% CI: 1.23-4.35; log-rank: *P* = 0.007) (Figure 2).

#### Time to first hospitalization for heart failure

**Figure 3:**
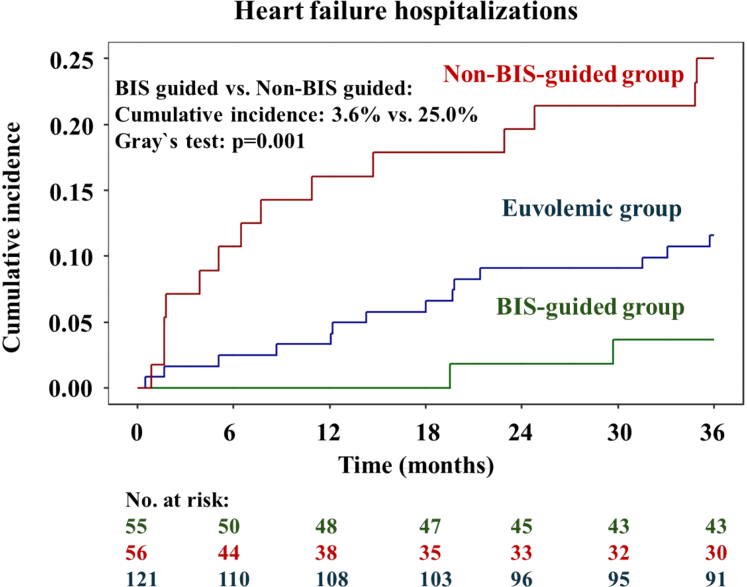
**Cumulative Incidence Functions Illustrating Differences in Time to First Heart Failure Hospitalization** Cumulative incidence functions demonstrating a 21.4% reduced cumulative incidence for time to first HFH, accounting for death as a competing risk, for BIS-guided vs non–BIS-guided patients. Abbreviation as in Figure 1.

At month 36, a total of 30 of 232 patients had experienced HFH, 2 of 55 patients in the BIS-guided group, 14 of 56 patients in the non–BIS-guided group, and 14 of 121 patients in the euvolemic control group. BIS-guided decongestion was associated with a 21.4% reduced cumulative incidence of first HFH compared to the non–BIS-guided group (3.6%; 95% CI: 0.7%-11.2% vs 25.0%; 95% CI: 14.5%-37.0%; Gray test: *P* = 0.001) (Figure 3). These results remained consistent in the per-protocol population (3.8%; 95% CI: 0.7%-11.6% vs 25.0%; 95% CI: 14.2%-37.4%; Gray test: *P* = 0.002) (Supplemental Table 3).

In the interventional period until 12 months, BIS-guided decongestion demonstrated a reduced cause-specific risk for time to first HFH (cumulative incidence: n.a. as 0 events). In a landmark analysis at 12-months, BIS-guided decongestion was associated with a nonsignificant 9.0% reduced cumulative incidence of the first HFH compared to the non–BIS-guided group (4.2%; 95% CI: 0.7%-12.7% vs 13.2%; 95% CI: 4.7%-26.0%; Gray test: *P* = 0.13).

Exploratory analysis demonstrated comparable cumulative incidence of the first HFH for patients in the BIS-guided group and the euvolemic control group (3.6%; 95% CI: 0.7%-11.2% vs 11.6%; 95% CI: 6.6%-18.0%; Gray test: *P* = 0.09). Conversely, patients in the non–BIS-guided group had a higher cumulative incidence of the first HFH than patients in the euvolemic control group (25.0%; 95% CI: 14.5%-37.0% vs 11.6%; 95% CI: 6.6%-18.0%; Grays test: *P* = 0.020) (Figure 3).

#### Frequency of hospitalization for heart failure

**Figure 4:**
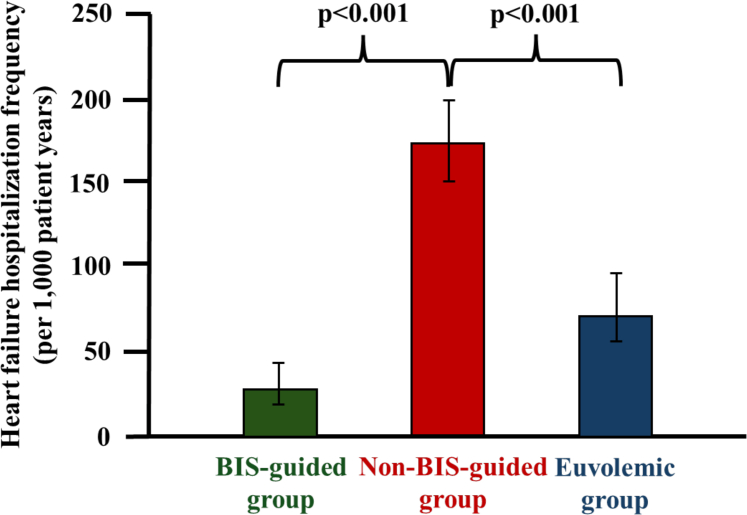
**Bar Charts Illustrating Differences in the frequency of Heart Failure Hospitalization** Bar charts demonstrate a reduced rate of HFH after 36 months for BIS-guided vs non–BIS-guided and vs euvolemic patients. Group differences were assessed using negative-binomial regression models. Abbreviation as in Figure 1.

After 36 months, a total of 49 HFH were recorded, 4 in the BIS-guided group, 22 in the non–BIS-guided group, and 23 in the euvolemic control group. The frequency of HFH was lower in the BIS-guided compared to the non–BIS-guided group (28 vs 173 per 1,000 patient-years; *P* < 0.001) (Figure 4). In the interventional period until 12-months, BIS-guided decongestion was associated with a reduced HFH frequency (0 vs 224 per 1,000 patient-years; *P* < 0.001). In a landmark analysis at 12 months, patients in the BIS-guided group had a lower HFH frequency than patients in the non–BIS-guided group (44 vs 141 per 1,000 patient-years; *P* = 0.043). These findings were confirmed using a negative binomial regression model. Model-based HFH rates were 0.028 (95% CI: 0.012-0.065) per patient year in the BIS-guided group compared to 0.188 (95% CI: 0.053-0.662) in the non–BIS-guided group, corresponding to a more than 6-fold higher HFH IRR for the non–BIS-guided group (IRR: 6.67; 95% CI: 2.61-17.09; *P* < 0.001).

Exploratory analysis demonstrated a lower HFH rate in the BIS-guided group compared to the euvolemic control group (28 vs 71 per 1,000 patient-years, *P* < 0.001) (Figure 4). Conversely, patients in the non–BIS-guided group had a higher HFH frequency than patients in the euvolemic control group (173 vs 71 per 1,000 patient-years, *P* < 0.001) (Figure 4).

All outcome measures are briefly described in Table 3 for the intention-to-treat population and in Supplemental Table 3 for the per-protocol population.

**Table 3:** Primary and Secondary Endpoints

Study Endpoint	BIS-Guided Group (n = 55)	Non–BIS-Guided Group (n = 56)	Measure of Effect
Primary endpoint			
HFH and/or all-cause death	12 (21.8%)	26 (46.4%)	HR: 0.40 95% CI: 0.20 to 0.79 log-rank: *P* = 0.006
HFH and/or all-cause death in the landmark analysis	5/48 (10.4%)	8/38 (21.1%)	HR: 0.47 95% CI: 0.15 to 1.44 log-rank: *P* = 0.18
Secondary endpoints			
All-cause death	11 (20.0%)	19 (33.6%)	HR: 0.54 95% CI: 0.26 to 1.14 log-rank: *P* = 0.10
All-cause death in the landmark analysis	4/48 (8.3%)	7/38 (18.4%)	HR: 0.57 95% CI: 0.16 to 2.03 log-rank: *P* = 0.39
HFH	2 (3.6%)	14 (25.0%)	Cumulative incidence: 3.6% vs 25.0% Gray test: *P* = 0.001
HFH in the landmark analysis	2/48 (4.2%)	5/38 (13.2%)	Cumulative incidence: 4.2% vs 13.2% Gray test: *P* = 0.13
Frequency of HFH	28/1,000 py	173/1,000 py	Negative-binomial regression model: 0.028/py vs 0.188/py *P* < 0.001

HFH = heart failure hospitalization; py = Patient-years; other abbreviation as in Table 1.

## Discussion

Decongestive treatment is well established in the management of patients with acute and chronic heart failure but has never been systematically assessed in the setting of AS.^14^, ^15^, ^16^, ^17^ EASE-TAVR was the first randomized, controlled trial to investigate the effects of targeted decongestive treatment following TAVR and demonstrated a reduction in the risk of HFH and/or all-cause death as well as an improvement in quality of life at 12 months after TAVR.^9^ Whether intensified diuretic treatment in AS with VO conveys prognostic benefits beyond 1 year is unknown. The present long-term extension data from EASE-TAVR shows for the first time that benefits derived from targeted and intensified diuretic treatment in the first 12 months after TAVR persist up to 3 years. These results corroborate the role of targeted decongestion as a treatment pillar in post-TAVR care.

In severe AS treated with valve replacement, the presence and prevalence of persistent heart failure following intervention has been widely neglected for a long time.^2^ More recently, the persistent heart failure component after valve replacement has been increasingly acknowledged with effectiveness of heart failure agents being investigated in this population.^18^^,^^19^ Dapagliflozin in Patients Undergoing Transcatheter Aortic-Valve Implantation (Dapa-TAVI) demonstrated a risk reduction for a composite endpoint of worsening heart failure and/or all-cause death at 12 months after TAVR.^18^ In Ramipril After Transcatheter Aortic Valve Implantation in Patients Without Reduced Ejection Fraction, renin-angiotensin system blockade post-TAVR resulted in a lower rate of heart failure readmissions at 1-year post-TAVR, even though the primary composite endpoint of reductions in cardiac mortality, heart failure readmission and stroke was not met.^19^ Similarly, in EASE-TAVR, intensified targeted decongestion reduced the composite of HFH and death post-TAVR.^9^ However, available outcome data regarding effectiveness of heart failure management strategies are currently restricted to 12-month follow-up. The present long-term extension analysis of EASE-TAVR for the first time provides outcome data beyond 1 year.

At 3 years post-TAVR, targeted decongestive treatment within the first year of TAVR resulted in higher diuretic doses over the 36-month follow-up period and in a 60% reduction in the hazard of HFH and/or all-cause death. In keeping with previous observations, this effect was driven by the reduction in the cumulative incidence of first HFH. However, as opposed to previous reports of heart failure management in AS, we also observed a trend toward mortality reduction in the BIS-guided decongestion group. This finding is remarkable, as valve replacement currently represents the only treatment which has been demonstrated to improve survival in AS whereas all other pharmacological interventions (either before or after valve replacement) have failed.

To investigate whether targeted decongestion conveys additional benefit beyond 1 year, landmark analyses were performed. Interestingly, despite no further study interventions being taken after 1 year, continued beneficial effects with respect to the composite of HFH and death was observed (ARR ∼10%) although the difference was not statistically significant. As numbers at risk beyond 1 year were small and the study was therefore not sufficiently powered for this landmark analysis, these results should be interpreted with caution. However, these findings are intriguing as clear further separation of event curves was seen beyond 1 year. In EASE-TAVR, diuretic doses were higher in the BIS-guided group throughout the entire trial, including the last study visit at 1 year. This intensified diuretic regimen established by the study team was upheld during continued follow-up, resulting in higher furosemide-equivalent doses in the BIS-guided vs the non–BIS-guided group through 36 months. During the first year, the study team also enforced patient self-management, self-care, and awareness for potential (re)occurrence of heart failure. Intensified decongestive treatment combined with rigorous patient education entailing an enduring behavioral effect likely conveyed continued benefits exceeding the 1-year study period. Accordingly, HFH frequency in the BIS-guided group was lower compared to the non–BIS-guided group and the euvolemic control group suggesting causative mechanisms beyond decongestion. Notably, previous data from retrospective cohort studies suggested that chronic diuretic use is associated with worse clinical outcomes, likely reflecting persisting congestion, and disease severity.^20^^,^^21^ Conversely, patients with VO in the BIS-guided vs the non–BIS-guided group in the present study had similar baseline risk profiles. Nevertheless, no fixed regimen was pursued in the BIS-guided group but treating physicians were encouraged to adjust diuretic treatment according to BIS measurement and target dry body weight—including dose reduction once euvolemia was reached. Therefore, higher diuretic doses in the BIS-guided group throughout the study likely reflected undertreatment of the non-BIS-guided group.

This study used BIS to assess VO, but other measures of objective volume status evaluation may be equally effective. Lung ultrasound, for instance, has been used successfully to evaluate pulmonary congestion in chronic heart failure patients which was associated with HFH and death.^22^, ^23^, ^24^ Decongestion guided by lung ultrasound compared to clinical judgment alone was demonstrated to improve clinical endpoints,^25^ and the potential of lung ultrasound in guiding the diuretic management in patients with heart failure has been widely acknowledged.^24^ Also, lung ultrasound may represent a more easily accessible and cheaper alternative for repetitive measurements in the context of longitudinal assessment.

With growing evidence for the importance of pharmacological interventions targeting the persistent heart failure component after AVR, the question of whether to combine respective agents will become of utmost importance. Studies involving cardiac magnetic resonance imaging have revealed that patchy scar induced by pressure overload in AS and depicted by late gadolinium enhancement is irreversible after AVR.^3^ However, diffuse fibrosis which demonstrates an endocardial to epicardial gradient may have the potential to reverse and therefore represent an attractive target for pharmaceutical interventions as an adjunct to valve replacement.^26^, ^27^, ^28^ Cardiac amyloidosis may also coexist with AS,^29^^,^^30^ may unfavorably influence reverse remodeling,^31^ and should therefore be recognized and treated to improve prognosis.^32^ The magnitude of additive treatment benefits derived from heart failure agents in post-TAVR care remains to be evaluated in future studies.

### Study Limitations

We acknowledge the limitation of a relatively small study size and low event counts. A qualitative fragility-style assessment indicated that modest changes in event counts could affect estimated treatment effects. However, ∼85% of the study population was still available for inclusion in the long-term extension period. Importantly, BIS-guided decongestion was not performed in the long-term extension phase beyond 1 year after TAVR. However, diuretic treatment was captured at yearly on-site follow-up visits and revealed persistently higher furosemide equivalent doses in the BIS-guided group throughout the trial phase and the long-term extension period until month 36 after TAVR. Unfortunately, data on trajectories of weight, N-terminal pro-B-type natriuretic peptide, Kansas City Cardiomyopathy Questionnaire, or renal function were not assessed systematically in the observational long-term extension period and were therefore not available. Yearly follow-up visits during the observational period were not mandatory and therefore not completed by all patients, potentially introducing attrition bias. Nevertheless, more than two-thirds of patients attended all scheduled follow-up visits and clinical outcome data were 100% complete. Importantly, prescription of heart failure medication of randomized patients with VO was well balanced between groups throughout the trial indicating that observed findings did not result from differences in adjunctive heart failure management, which is corroborated by consistent results after adjustment for heart failure medication.

## Conclusions

The present long-term extension study of EASE-TAVR is the first to provide long-term clinical outcome data of heart failure management after TAVR. Targeted diuretic treatment within the first year of TAVR combined with rigorous patient education led to higher diuretic doses throughout the observational long-term extension period and to early clinical improvements which were maintained and enhanced over a period of 36 months. This included significant reductions in the composite of HFH and death, and HFH alone, as well as a nonsignificant reduction in mortality hazard, which is unique for this population. Exploratory analysis indicated that BIS-guided decongestion reduced heart failure and mortality hazard to the level of euvolemic controls. We conclude that individualized decongestive treatment should be considered in post-TAVR care to improve outcomes, until a larger multicenter trial disputes these findings.

Perspectives **COMPETENCY IN MEDICAL KNOWLEDGE:** The present long-term extension study of EASE-TAVR is the first to provide long-term clinical outcome data of heart failure management after TAVR. We show that targeted decongestive treatment leads to early clinical improvements that are maintained and enhanced over a period of 3 years resulting in a 60% reduced hazard in the composite of HFH and all-cause mortality compared to decongestion by clinical judgment alone. This effect was mainly driven by a reduction in the cumulative incidence of first HFH, but we also observed a nonsignificant reduction in all-cause mortality, which is unique for heart failure management in this patient population.

**TRANSLATIONAL OUTLOOK:** Individualized decongestive treatment should be considered in post-TAVR care to improve outcomes, until a larger multicenter trial disputes these findings.

## Funding support and author disclosures

Dr Nitsche has received speaker/consulting honoraria from Pfizer, Bayer, Prothena, and Böhringer Ingelheim and research contracts with Pfizer, AstraZeneca, the Austrian Society of Cardiology, the European Association of Cardiovascular Imaging, and the Austrian Science Fund. All other authors have reported that they have no relationships relevant to the contents of this paper to disclose.

## Supplementary material

Supplementary docx 1
