# Supplementary material for: Long-Term Outcomes of Targeted Volume Overload Management in Patients With Severe Aortic Stenosis
Source: JACC Adv. 2026 Apr 1;5(5):102705. doi: 10.1016/j.jacadv.2026.102705 (PMC13087758; doi:10.1016/j.jacadv.2026.102705)
Supplement: Supplementary docx 1 [file mmc1.docx]

**Long-term outcomes of targeted fluid overload management in patients with severe aortic stenosis.**

**Supplementary files**

**Supplementary Figure 1.** CONSORT flow diagram of the study population.


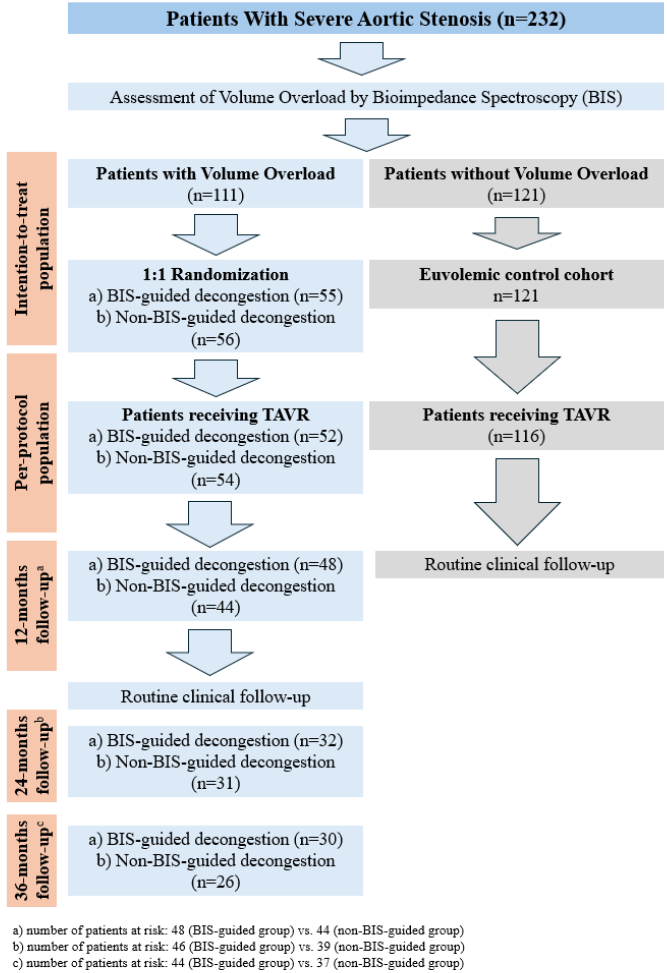


**Supplementary Table 1.** Heart failure- and diuretic medication at randomization.

|  | **Missing values** | **Volume Overload**  **(n=111)** | | **No Volume Overload/**  **Euvolemic control group**  **(n=121)** |
| --- | --- | --- | --- | --- |
|  |  | **BIS-guided group**  **(n=55)** | **Non-BIS-guided group**  **(n=56)** |  |
| **Heart failure medication** |  |  |  |  |
| Any RAS inhibitor, % | 12/232 | 29 (65.9) | 37 (66.1) | 70 (58.3) |
| ACE inhibitor, % | 12/232 | 16 (36.4) | 11 (19.6) | 34 (28.3) |
| ARB, % | 12/232 | 11 (25.0) | 22 (39.3) | 34 (28.3) |
| ARNI, % | 12/232 | 3 (6.8) | 4 (7.1) | 2 (1.7) |
| MRA, % | 11/232 | 11 (25.0) | 19 (33.9) | 33 (27.3) |
| Beta-blocker, % | 12/232 | 24 (54.5) | 34 (60.7) | 69 (57.5) |
| SGLT2 inhibitor, % | 17/232 | 5 (12.2) | 5 (8.9) | 21 (17.8) |
| **Diuretic medication** |  |  |  |  |
| Any diuretic drug, % | 12/232 | 28 (65.1) | 38 (67.9) | 60 (49.6) |
| Loop diuretic, % | 11/232 | 20 (45.5) | 32 (57.1) | 46 (38.0) |
| Thiazide, % | 11/232 | 9 (20.5) | 4 (7.1) | 4 (3.3) |

Categorical variables are presented as count (percentage) and continuous variables are reported as median (25-75^th^ percentile).

ACE, indicates Angiotensin converting enzyme; ARB, Angiotensin II receptor blocker; ARNI, Angiotensin receptor neprilysin inhibitor; BIS, Bioimpedance spectroscopy; HF, Heart failure; MRA, Mineralocorticoid receptor antagonist; RAS, Renin angiotensin system; SGLT2, Sodium glucose co-transporter 2

**Supplementary Table 2.** Primary endpoint after adjustment for HF medications in the overall- and in the landmark analysis.

|  | **Hazard Ratio** | **95% CI** | **P-Value** |
| --- | --- | --- | --- |
| **HFH and/or all-cause death at month 36** |  |  |  |
| -Unadjusted | 0.40 | 0.20 to 0.79 | 0.006 |
| -Adjusted for number of HF medications | 0.41 | 0.21 to 0.81 | 0.013 |
| -Adjusted for any RAS inhibitor | 0.40 | 0.20 to 0.80 | 0.008 |
| -Adjusted for MRA | 0.39 | 0.20 to 0.78 | 0.007 |
| -Adjusted for Beta-blocker | 0.40 | 0.20 to 0.80 | 0.009 |
| -Adjusted for SGLT2 inhibitor | 0.40 | 0.20 to 0.79 | 0.009 |
| **HFH and/or all-cause death in the landmark analysis** |  |  |  |
| -Unadjusted | 0.47 | 0.15 to 1.44 | 0.18 |
| -Adjusted for number of HF medications | 0.45 | 0.14 to 1.39 | 0.16 |
| -Adjusted for any RAS inhibitor | 0.47 | 0.15 to 1.43 | 0.18 |
| -Adjusted for MRA | 0.48 | 0.16 to 1.46 | 0.20 |
| -Adjusted for Beta-blocker | 0.49 | 0.16 to 1.50 | 0.21 |
| -Adjusted for SGLT2 inhibitor | 0.44 | 0.14 to 1.36 | 0.16 |

Unadjusted hazard ratios were derived from univariable Cox proportional hazard regression models and between-group comparisons were performed using the log-rank test. Adjusted hazard ratios and corresponding p-values were derived from multivariable Cox proportional hazards regression models.

CI, indicates Confidence interval; HF, Heart failure; HFH, Heart failure hospitalization; MRA, Mineralocorticoid receptor antagonist; RAS, Renin-angiotensin system; SGLT2, Sodium-glucose co-transporter 2

**Supplementary Table 3:** Primary and secondary endpoints in the per-protocol population.

|  | **BIS-guided group (n=53)** | **Non-BIS-guided group (n=52)** | **Measure of effect** |
| --- | --- | --- | --- |
| **Primary Endpoint** |  |  |  |
| HFH and/or all-cause death | 11 (20.8%) | 22 (42.3%) | HR: 0.42  95% CI: 0.21 to 0.87  log-rank: p=0.023 |
| HFH and/or all-cause death in the landmark analysis | 5/47 (10.6%) | 7/37 (18.9%) | HR: 0.55  95% CI: 0.17 to 1.72  log-rank: p=0.30 |
| **Secondary Endpoints** |  |  |  |
| All-cause death | 10 (18.9%) | 16 (30.8%) | HR: 0.57  95% CI: 0.26 to 1.25  log-rank: p=0.16 |
| HFH | 2 (3.8%) | 13 (25.0%) | Cumulative incidence:  3.8% vs. 25.0%  Gray`s test: p=0.002 |
| Frequency of HFH | 29/1000 py | 172/1000 py | Negative-binomial regression model:  0.029/py vs. 0.190/py  p=0.001 |

BIS, indicates Bioimpedance spectroscopy; CI, Confidence interval; HFH, Heart failure hospitalization; HR, Hazard ratio; py, Patient years

**Supplementary Table 4:** Comparison of BIS-guided vs. euvolemic patients after multivariable adjustment for EuroSCORE-II, NT-proBNP and eGFR.

|  | **Hazard Ratio** | **95% CI** | **P-Value** |
| --- | --- | --- | --- |
| **Primary endpoint** |  |  |  |
| HFH and/or all-cause death at month 36 | 0.84 | 0.42 to 1.65 | 0.61 |
| HFH and/or all-cause death in the landmark analysis | 0.62 | 0.22 to 1.75 | 0.37 |
| **Secondary endpoints** |  |  |  |
| All-cause death | 1.22 | 0.58 to 2.59 | 0.60 |
| HFH | 0.29 | 0.07 to 1.31 | 0.11 |

Adjusted hazard ratios and corresponding p-values were derived from multivariable Cox proportional hazards regression models.

BIS, indicates Bioimpedance spectroscopy; CI, Confidence interval; eGFR, Estimated glomerular filtration rate; EuroSCORE-II, European System for Cardiac Operative Risk Evaluation; HFH, Heart failure hospitalization; NT-proBNP; N-terminal pro-B-type natriuretic peptide
